# Novel Missense Mutation in the *NOD2* Gene in a Patient with Early Onset Ulcerative Colitis: Causal or Chance Association?

**DOI:** 10.3390/ijms15033834

**Published:** 2014-03-03

**Authors:** Martina Girardelli, Josef Vuch, Alberto Tommasini, Sergio Crovella, Anna Monica Bianco

**Affiliations:** 1 Institute for Maternal and Child Health—IRCCS “Burlo Garofolo”, Via dell’Istria 65/1, Trieste 34137, Italy; E-Mails: alberto.tommasini@burlo.trieste.it (A.T.); sergio.crovella@burlo.trieste.it (S.C.); annamonicarosaria.bianco@burlo.trieste.it (A.M.B.); 2 Department of Medicine and Surgery and Health, University of Trieste, Piazzale Europa, 1, Trieste 34128, Italy; E-Mail: josef.vuch@burlo.trieste.it

**Keywords:** complex disease, digenic heterozygosis, early onset-inflammatory bowel disease, *IL10R*, *NOD2*

## Abstract

Deregulated immune response to gut microflora in genetically predisposed individuals is typical for inflammatory bowel diseases. It is reasonable to assume that genetic association with the disease will be more pronounced in subjects with early onset than adult onset. The nucleotide-binding oligomerization domain containing-2 gene, commonly involved in multifactorial risk of Crohn’s disease, and interleukin 10 receptor genes, associated with rare forms of early onset inflammatory bowel diseases, were sequenced in an early onset patient. We identified a novel variant in the *NOD2* gene (c.2857A > G p.K953E) and two already described missense variants in the *IL10RA* gene (S159G and G351R). The new *NOD2* missense variant was examined *in silico* with two online bioinformatics tools to predict the potentially deleterious effects of the mutation. Although cumulative effect of these variations in the early onset of the disease can be only hypothesized, we demonstrated that family information and *in silico* studies can be used to predict association with the disease.

## 1. Introduction

Inflammatory bowel diseases (IBDs) are chronic inflammatory disorders of intestine that result from a deregulated mucosal immune response in genetically predisposed individuals [1]. It is a widespread pathological condition with increased incidence in Western populations, affecting approximately one out of 250 people. The two major forms of IBD, Crohn’s disease (CD) and ulcerative colitis (UC), are usually quite distinct. Although the age of onset of IBD is usually in early adulthood, the pathology could also occur in childhood during the first years of life. Early onset IBD patients often present overlapping features of CD and UC, making a definite diagnosis difficult. Various epidemiological studies suggest that patients with an early onset diagnosis usually manifest a more serious and aggressive course, tending to a very fast development [2]. Evidence supports the hypothesis that genetic predisposition plays a more substantial role in pediatric patients, who manifest a more extensive and severe course of the disease than subjects with adult onset.

Several genes, identified by several genome-wide association studies (GWAS), have been associated with IBD predisposition, both in adult and early onset. In addition, a number of monogenic immune disorders have been found in a small, but increasing, portion of early onset IBD, suggesting that common genetic variants and rare monogenic defect can play a synergic and crucial role in the development of the disease.

Considering that GWAS could only explain about half of the genetic heritability of IBD, it has been suggested that the additive role of multiple variants and/or rare “private” genes not identified by GWAS may account for the so-called “missing heritability”; this could be even more likely for early onset IBD, in which higher genetic risk is expected.

The patient we are going to describe is a child (present age: seven years) presenting a very early onset ulcerative colitis (VEO-UC), cared for at the Pediatric Gastroenterological Service of the Institute for Maternal and Child Health IRCCS “Burlo Garofolo” (Trieste, Italy): at 18 months, pancolitis with mucosal and intracryptic localization was diagnosed. No extra-intestinal manifestations and/or associated diseases were present. At colonoscopy, rectal inflammation with hemorrhagic ulcers and mucosal edema were evidenced. The colonic mucosa was very fragile and tended to bleed easily. Intestinal biopsies showed chronic inflammation with lymphocytic aggregates. The search for granuloma and viral inclusions results as being negative. The disease showed a severe course in spite of the use of glucocorticoids, azathioprine and thalidomide administration. A good clinical response was achieved only with a biological therapy with infliximab (anti-TNF-α antibodies). In order to assess monogenic causes of early onset inflammatory colitis in this patient, we analyzed both subunits alpha and beta of the interleukin-10 receptor (*IL10RA* and *IL10RB*), as well as nucleotide-binding oligomerization domain containing 2 (*NOD2*), since these genes are known to be associated with a higher risk for CD.

## 2. Results and Discussion

### 2.1. Results

**Figure 1.**
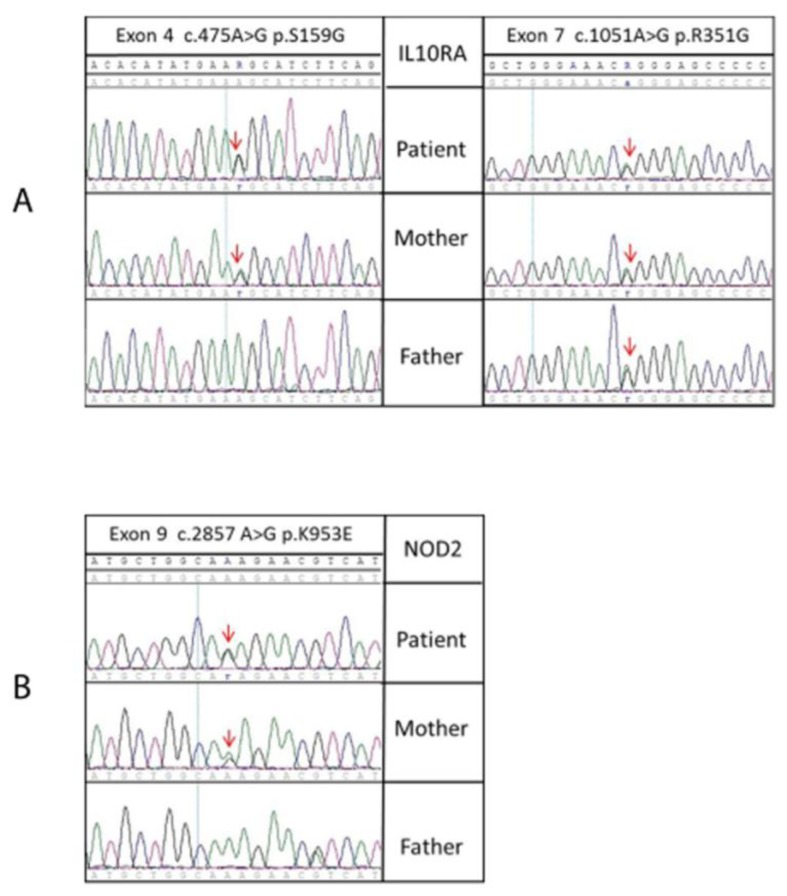
Sequence chromatograms from patient and parents of the *IL10RA* and *NOD2* coding regions. (**A**) In the *IL10RA* exon 4 and exon 9, the arrows indicate the nucleotide substitution, c.475A > G and c.1051A > G, consisting, respectively, in the amino acid substitutions, S159G (A/G heterozygous patient and mother, A/A wild type father) and R351G; (**B**) in the *NOD2* exon 9 sequence, the c.2857 A > G substitution consisted in an amino acid substitution, K953E (A/G heterozygous patient and mother, A/A wild-type father).

We found 18 variants in our patient, five in the *NOD2*, four in the *IL10RA* and nine in the *IL10RB* genes. All variants localized respectively at the 5′ and/or 3′ untranslated, intronic and coding regions (Table 1). Among the variants identified in *NOD2*, four are known variants, and one, is a novel missense variant at the exon 9 (c.2857A > G p.K953E) present in heterozygosis (Figure 1B). Within the three variants in the coding sequence of *IL10RA*, two missense variants, both present in heterozygosis, rs3135932 (c.475A > G p. S159G) and rs2229113 (c.1051 G > A p.G351R), have already been described in the literature.

The three-missense variants were searched for in both parents to check their pattern of inheritance. The mother carried the three variants (K953E, S159G and G351R) observed in the patient, while the father results heterozygous only for the G351R variant (Figure 1). The new *NOD2* missense variant was searched for in 60 anonymous healthy Italian donors to confirm if it was not a polymorphism, but a true mutation: no variant has been found.

**Figure 2.**
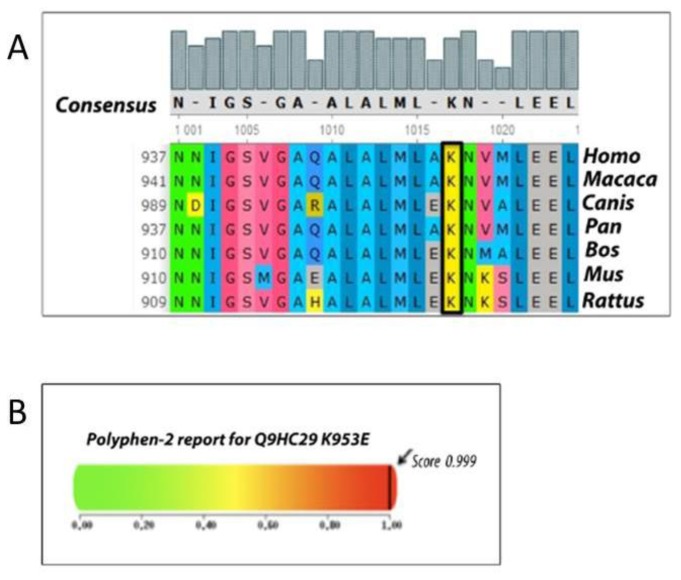
Bioinformatics analysis results. (**A**) Multiple alignment of the amino acid sequence of *NOD2* protein in seven species showed that this is a conserved region; (**B**) PolyPhen2 (Polymorphism Phenotyping v.2) analysis predicting the probably damaging impact of the K953E substitution with a score of 0.999.

All variants were already described in the common SNPs Database (Single Nucleotide Polymorphisms at http://www.ncbi.nlm.nih.gov/snp/), as polymorphic in the general population, except for the new *NOD2* variant, for which we performed an *in silico* analysis with PolyPhen2 (Polymorphism Phenotyping v.2) and SIFT (Sorting Intolerant from Tolerant). Both online tools showed a close agreement in the scores, predicting this new missense variant, K953E, as probably damaging. Several alignments in different species showed that this is a conserved region (Figure 2).

### 2.2. Discussion

In our case report study, we analyzed a set of three genes, described as strongly associated with IBD [3,4], in a child affected by severe early onset inflammatory colitis. Among the 18 variants, three missense variations, the new *NOD2* mutation (K953E) and the known *IL10RA* missense variants (S159G and G351R), caught our attention: we hypothesized that these three variants might together contribute to an increased risk of developing early onset IBD [5]. Variants in combined heterozygosis on different genes may have a cumulative effect and contribute to the disease phenotype, probably due to the dysregulation of different pathways. Digenic contribution to multigenic diseases is poorly investigated by GWAS studies. However, recent data showed that digenic or multigenic inheritance might be underrated mechanisms explaining familial recurrence of multifactorial diseases [6].

*IL10RA* (11q23) and *IL10RB* (21q22) are attractive inflammatory bowel disease candidate genes: they encode for the alpha and beta chain, respectively, that form a tetrameric cell bound structure able to bind interleukin (IL-10). The IL-10 pathway is very important in regulating the intestinal inflammation, and mutations in the receptor cancel the IL-10 immunomodulatory signal, which is strongly associated with an intestinal hyper-inflammation [7]. Although there are no data about the association between these variants (S159G and R351G) and the early onset of the disease, several studies have shown a loss of function due to these variants [8,9].

*NOD2*, localized at chromosome 16q21, codify for a protein that belongs to the family of intracellular NLR (NOD-like receptors), able to recognize microbial components and to stimulate an inflammatory response through the activation of NF-κB. Moreover, three *NOD2* mutations (rs2066844, rs2066845 and rs5743293) represent the main genetic factor causing susceptibility to CD. These mutations occur in the LRR domain or in the neighboring domain, suggesting that they alter the receptor capacity to recognize the bacterial component [10].

Although the new K953E mutation in *NOD2* has not been previously reported, it is likely to disrupt *NOD2* protein function for several reasons: (1) the mutation is a disfavored substitution of an evolutionarily conserved leucine residue repeat; (2) the mutation occurs in the LRR domain, suggesting that it could alter the recognition of the bacterial agent; (3) the amino acid, K, is highly conserved and the resulting amino acid change was predicted to be deleterious by both SIFT [11] and PolyPhen2 software [12,13] (Figure 2). However, also the mother of the IBD affected child was heterozygous for this mutation, not presenting, nevertheless, any disease signs. Therefore, even if *in silico* analysis considered the K953E *NOD2* mutation as harmful, we are aware that further studies are needed to elucidate the importance of this variant in the setting of early severe colitis.

We can speculate that the new mutation in the *NOD2* gene described in our early-onset UC patient could alter the normal function of the receptor that plays a central role in the pro-inflammatory response and bacterial sensing. The other two variants in the subunit alpha of the *IL10R* are responsible for a decreased activity of the receptor. Therefore, our assumption is that, although these variations are located on two different genes and apparently belong to different molecular pathways, they could somehow determine a cumulative dysfunctional effect, causing themselves the peculiarities of this form of ulcerative colitis.

We are aware that these are just hypotheses that should be confirmed by functional studies, lacking in this case report and representing a limit of our study. Moreover, the presence of *NOD2* (K953E) and *IL10RA* variants in the healthy mother suggests that the associated risk may not be so high. This brings us back to the concept of multifactorial disease in which also the non-genetic factor should be considered: for this reason, we can therefore hypothesize that the disease exhibited by the child could be given by the combination of the three variants with an additional factor, such as diet, antibiotic consumption and microbiome, and other not yet investigated genes. Thus, further analyses, including functional studies to verify the true effect of *NOD2* mutation and *IL10RA*, should be performed, recruiting a larger number of patients in order to better understand the genotype-phenotype correlation, in relation to an additional factor or an additional gene.

## 3. Experimental Section

### 3.1. DNA Extraction and Genotyping

Genomic DNA was extracted from 1–2 mL EDTA-anticoagulated blood using an EZ1 DNA Blood Kit (QIAGEN, Milano, Italy), according to manufacturer’s instructions. PCR amplification was performed for the entire coding sequence, flanking regions and for 5′ and 3′ untranslated region of the *NOD2* and *IL10R* genes, using the KAPA 2G Fast Hot Start Readymix (RESNOVA, Roma, Italy) and using a 2720 Thermal Cycler (Applied Biosystems, Foster City, CA, USA). Amplification products were directly sequenced by the Sanger method using an ABI PRISM 3130XL automated DNA sequencer (Applied Biosystems, Foster City, CA, USA). Sequences were analyzed with Seqman II Software (DNASTAR I Lasergene, 7.0, Madison, WI, USA). While primers used for amplification of IL10 receptor genes (IL10RA and IL10RB) were designed according to the indications of Glocker *et al.* [3]; *NOD2* primers used both for PCR and sequencing were designed using Primer Blast based on the relative sequence deposited in GenBank: NM_022162 (*NOD2/CARD15*). Each variant has been identified and verified in databases by performing a nucleotide BLAST (Basic Local Alignment Search Tool) server (http://blast.ncbi.nlm.nih.gov/).

### 3.2. *In Silico* Analysis

The bioinformatics analysis of missense variants was performed with two online tools that can predict the possible impact of an amino acid substitution on both the structure and function of a human protein using straightforward physical and comparative considerations: PolyPhen2 (version 2.2.2, PMID: 20354512 and 23315928, Boston, MA, USA), (http://genetics.bwh.harvard.edu/pph2/) and SIFT (version 1.03, PMID: 11337480 Seattle, WA, USA), (http://sift.jcvi.org).

## 4. Conclusions

Finally, in the context of this kind of genetic disease, we would like to emphasize that genetic analyses of many genes can open new scenarios that could be unraveled only by large studies and robust biologic and bioinformatics evaluations. For this reason, a new generation sequencing (NGS) approach must be taken into account to seek an alternative genetic cause that may contribute to the disease phenotype.

## Figures and Tables

**Table 1.** Genetic variant in the analyzed genes, relative genotyping and localization.

Gene	Variants		Status	Site
*NOD2*	rs5743266	c.-59G > A	A/A	5′UTR
	rs2076753	c.74 - 25G > T	T/T	Intron 1
	rs2066842	c.802C > T p.P268S	T/T	Exon 4
	rs2066843	c.1377C > T p.R459R	T/T	Exon 4
	NEW	c.2857A > G p.K953E	A/G	Exon 9

*IL10RA*	rs10892202	c.67 + 89G > C	G/C	Intron 1
	rs4252249	c.180G > A p.A60A	G/A	Exon 2
	rs3135932	c.475A > G p.S159G	A/G	Exon 4
	rs2229113	c.1051A > G p.R351G	A/G	Exon 7

*IL10RB*	rs2239573	c.49 + 40G > A	G/A	Intron 1
	rs2843701	c.498 + 234C > T	T/T	Intron 4
	rs71973425	c.804 + 188_804 + 189ins AGGGAAGTCTG	ins/ins	Intron 6
	rs2276223	c.804 + 234T > G	G/G	Intron 6
	rs2247878	c.804 + 240T > C	C/C	Intron 6
	rs2507737	c.804 + 335A > G	G/G	Intron 6
	rs8178528	c.804 + 414A > G	G/G	Intron 6
	rs8178529	c.804 + 421A > C	C/C	Intron 6
	rs8178561	c.*135G > A	G/A	3′UTR
